# Investigation of serum level relationship of pro-inflammatory and anti-inflammatory cytokines with vitamin D among healthy Ghanaian population

**DOI:** 10.1186/s13104-024-06721-y

**Published:** 2024-03-04

**Authors:** Maxwell Hubert Antwi, Samuel Asamoah Sakyi, Seth Christopher Yaw Appiah, Tonnies Abeku Buckman, Joseph Yorke, Aaron Siaw Kwakye, Isaac Darban, Peter Agoba, Akwasi Minnah Addei

**Affiliations:** 1 Department of Molecular Medicine, School of Medicine and Dentistry, Kwame Nkrumah University of Science and Technology, Kumasi, Ghana; 2 Department of Medical Laboratory Science, Faculty of Health and Allied Sciences, Koforidua Technical University, Koforidua, Ghana; 3 Department of Sociology and Social Work, Kwame Nkrumah University of Science and Technology, Kumasi, Ghana; 4 Department of Medical Laboratory Science, KAAF University College, Buduburam, Ghana; 5 Department of Surgery, School of Medicine and Dentistry, Kwame Nkrumah University of Science and Technology, Kumasi, Ghana; 6 Department of Biochemistry, Kwame Nkrumah University of Science and Technology, Kumasi, Ghana; 7 Department of Biological Sciences, Kwame Nkrumah University of Science and Technology, Kumasi, Ghana

**Keywords:** 25(OHD) deficiency, TNF-alpha, IFN-gamma, IL 10, Blood donors, Ghana

## Abstract

**Background:**

The interplay between vitamin D status and inflammatory cytokines in a supposedly sufficient sunshine environment has not well been evaluated. The study sought to determine their association.

**Methods:**

This cross-sectional study involved 500 healthy adult blood donors from some selected hospitals in Ghana enrolled from June to November 2016. Venous blood samples were obtained from participants, 25(OH)D, TNF-alpha, IFN-gamma, and IL 10 were measured using enzyme linked immunosorbent assay (ELISA) technique. Serum levels of 25(OH)D < 20ng/ml were classified as being deficient or low.

**Results:**

The average age of the participants was 27.97 years. No statistically significant association was established between 25(OH) D status, mean age (*p* = 0.1693), and gender (*p* = 0.5461) of study participants. Similarly, the median 25(OH) D (*p* = 0.8392), IL-10 (*p* = 0.5355), TNF-alpha (*p* = 0.9740), and IFN-gamma (*p* = 0.6908) were not significantly different across gender. There was a significantly increased levels of TNF-alpha (*p* < 0.0001) and IFN-gamma (*p* < 0.0001) among participants with 25(OH) D deficiency compared to those without deficiency. Concurrently, participants with 25(OH)D deficiency had a significantly reduced levels of IL-10 (*p* < 0.0001) compared to those without 25 (OH) D deficiency. The most accurate biochemical markers for identifying 25 (OH) D deficiency were IFN-gamma (AUC = 0.879; *p* < 0.0001) followed by TNF-gamma (AUC = 0.849; *p* < 0.0001) and IL-10 (AUC = 0.707; *p* < 0.0001).

**Conclusion:**

There was a significant association between vitamin D levels and pro-inflammatory cytokines (TNF-alpha, IFN-gamma) and anti-inflammatory cytokine (IL 10) among healthy Ghanaian populace.

## Introduction

Vitamin D is produced from sunlight through skin exposure (about 90%) and also from diet (about 10%). Vit D produced from sunlight through skin exposure and the Vit D produced from diet is converted into 25-hydroxyvitamin D (25(OH)D) (the inactive form) in the liver and is carried into circulation by vitamin D binding protein to the kidney. The kidney then converts (25OHD) into the active form called 1, 25-dihydroxyvitamin D (1,25(OH)2D) through the activity of the enzyme 1-alpha hydroxylase (CYP27B1) whose function is dependent on parathyroid hormone (PTH), calcium and phosphorous levels [1]. The role of vitamin D has been observed in the immune functioning of the body aside from its conventional role in calcium and bone homeostasis. Studies on vitamin D and the immune system have been conducted and subjects with vitamin D deficiency remain highly susceptible to infection with low immunity [2, 3]. Previous studies have indicated that vitamin D is a pivotal regulator of immune function and inflammation and it is very essential in preventing some cancers [4]. Many tissues aside kidney, intestine, and skeletal express the Vitamin D Receptor gene (VDR) and 1-α-hydroxylase (CYP27B1) and have the ability to change 25OHD (the inactive form) to 1,25OHD (the active form) in a non-renal environment [5–7]. Immune cells and cells capable of causing cancer have been included in the tissues that express VDR and 1-α-hydroxylase (CYP27B1) and is an indication that apart from the major role of vitamin D in calcium and bone homeostasis, the hormonal form of vitamin D, 1,25- dihydroxyvitamin D3 influences the differentiation and role of both the innate and adaptive immune cell types and to further enhance cytokine production distinctively [8, 9].

Some of the non-conventional activities of vitamin D involve cell growth and distinction in addition to its effect on the immune response emerging in preparedness to support tolerance and to stimulate or advance defensive immunity [10, 11].Macrophages, dendritic cells, T and B cells all have the essential mechanism to produce and react to 1,25 OHD thereby making vitamin D act in either a paracrine or autocrine mode. The non-renal 1-α-hydroxylase enzyme in monocytes differ from the kidney one because its activity is not stimulated by parathyroid hormone. It rather depends on 25OHD (the inactive form) which is the major circulating form of vitamin D or pro-inflammatory cytokines such as IFN-γ, IL-6, or TNF-α induction which leads to vitamin D (1,25OHD) production [12, 13].

The effect of high vitamin D (1,25OHD) production subdues T cell growth by switching from a Th1 to a Th2 phenotype which decreases the production of inflammatory cytokines (TNF-α, IFN-γ, IL-17, IL-21) with high making of anti-inflammatory immune markers such as IL-10 [14, 15]. Present corroboration suggest that the circulating level of 25(OH)D may be pivotal for the ideal anti-inflammatory response of human monocytes [16]. The active metabolite of vitamin D (calcitriol) produced enhances the expression of anti-inflammatory cytokine like IL10 whose function is to inhibit other cytokines particularly the pro-inflammatory cytokines including TNF-α, IFN-γ, IL-17, and IL-21. This is capable of causing tissue damage and diseases [12, 17]. The active form of vitamin D 1,25(OH)2D plays a very essential role in calcium and phosphorous homeostasis and gives vitamin D the endocrine, autocrine, and paracrine role by acting on related tissues responsive to vitamin D [18].

Notwithstanding, the interplay between vitamin D status, inflammatory cytokines, and immune cells in the supposedly sufficient sunshine environment has received minimal evaluation. Most studies conducted that have attempted to explore the association between vitamin D status and cytokines in Africa have not been carried out among apparently healthy individuals. There is paucity of studies on the association between vitamin D status and pro-inflammatory and anti-inflammatory cytokines among the Ghanaian populace. This study aims to explore the association between vitamin D status, inflammatory cytokines and immune cells in apparently healthy Ghanaian population.

## Materials and methods

### Study design/ settings

This cross-sectional study was conducted in seven randomly selected hospitals across seven regions of Ghana from June to November 2016. Four (4) district hospitals which included St. Patrick’s hospital Offinso-Maase-Ashanti Region (AR), Nandowli hospital-Upper West Region (UWR), Kpandai hospital-Northern Region (NR), and Wenchi Methodist hospital-Brong-Ahafo (BA) were selected. Three (3) regional hospitals which included; Bolgatanga Regional hospital-Upper East Region (UER) Effia Nkwanta Regional hospital-Western Region (WR), and Koforidua Regional hospital-Eastern Region (ER) were also selected. Based on the bed capacity, attendance rate, and referral policy, 100 participants were selected from each regional hospitals and 50 blood donors each were also selected from the various district hospitals. Ghana is geographically placed near the equator and lies between latitudes of 4°N and 12°N, and longitudes of 4°W and 2°E. It occupies an area of about 240,000 km^2^ of the African continent and is the thirteenth most populated country in Africa, and among the top five populated countries in West Africa, with more than 26 million people accounting for about 2.5% of African’s population.

### Study population/ subjects selection

Using simple random and systematic sampling technique, a total of five hundred (500) healthy blood donors between the ages of 17 and 60 years for males and 17 to 45 years for females were selected. The blood donors consist of those from blood mobilizations drive in schools and churches as well as those who visit the blood bank clinics for donations. Those who qualified and safe for blood donation at the various selected hospital blood bank units across seven regions of Ghana were recruited for this study.

### Inclusion criteria

Healthy blood donors who have passed screening and were free from any diseases including pulmonary tuberculosis, diabetes mellitus, malaria, sexually transmitted infections (STIs), and blood borne infections were included in this study.

### Exclusion criteria

Blood donors with clinical records or symptoms showing chronic ailments such as renal, hypertension and any other diseases, subjects with drugs that could influence vitamin D intake and metabolism were all excluded from the study. Donors who did not give consent to be part of the study were equally excluded.

### Sample collection and processing

Ten (10) milliliters (ml) of venous blood were taken from prominent vein of each donor. Four (4) ml of blood were dispensed into dipotassium ethylene diamine tetra acetic acid (K_2_EDTA) tube, one (1) ml into Fluoride oxalate tubes and five (5) ml into gel separator tubes (5 ml MICROPOINT clot activator tube; batch number: KJ040AS). K_2_EDTA blood samples were screened for haemoglobin levels, typhoid test, hepatitis B and C, HIV/AIDs, syphilis, and malaria. Calibrated copper solution of standard hemoglobin concentration of 13.0 g/dl and 12.5 g/dl for males and females respectively was the method used to determine the hemoglobin level of the blood donors. The tubes were positioned in the centrifuge holes (HERMLE Z300K, Labsource, Inc. Romeoville, IL 60,446) and spun at 3500 rpm for about 8 min to get the plasma and serum where appropriate. Blood collected into the fluoride oxalate tubes was screened for diabetes. The serum was aliquoted into cryo-tubes and stored at − 70 °C (Thermo Scientific™ Revco™ UxF − Ultra-Low Temperature Freezers, USA) until the measurement of the biochemical assays.

### Biochemical assays

25-Hydroxy [25(OH)D] and the cytokines (IL10, TNF-alpha, IF-gamma) were purchased from Biobase Biotech (Jinan) Co., Ltd, China, and Biolegend Co., Ltd USA respectively. The reagent from Biobase Biotech (Jinan) Co., Ltd, China, and Biolegend Co., Ltd USA whose basic principle is enzyme linked immunosorbent assay (ELISA) were employed to determine the concentrations of 25(OH)D and IL10, TNF-alpha, IFN-gamma respectively of all the study participants as stated by the producer’s protocol. Their various absorbances were then measured spectrophotochemically using Inqaba biotec ELISA plate reader (Inqaba Biotechnical Industries (Pty) Ltd, South Africa).

### Classification of [25(OH)D status

25(OH)D serum concentrations were classified into extreme deficiency, not extreme or risk of deficiency, optimal (ideal), high risk, and harmful levels as follows; < 10ng/ml, 10-19ng/ml, 20-50ng/ml, 51-80ng/ml and > 80ng/ml respectively [19]. Eventually, every 25(OH)D levels less than 20ng/ml were deemed to be vitamin D deficient and greater than 20ng/ml as not deficient.

### Statistical analysis

The Statistical Package for the Social Sciences (SPSS) version 20 (SPSS Inc., Chicago, USA) was used to generate and analyze data and cleaned for outrageous values at regular intervals. Categorical variables were presented and reported as frequencies with their corresponding percentages. Means with their standard deviation and median (ranges) were used to summarize continuous variables. Comparison between two means of both parametric and non-parametric continuous variables was done using the Unpaired t-test and Mann-Whitney U test respectively. The chi-square test statistic was employed to determine any correlation between the categorical variables. Spearman’s rho statistic was used to determine the relationship between vitamin D (25OHD) and the biochemical parameters while the odds of biochemical variables in predicting vitamin D deficiency were determined using multivariate logistic regression analysis. A p-value less than 0.05 was considered statistically significant. Coefficients were then determined with their respective 95% confidence intervals.

## Results

The table shows socio-demographics characteristics stratified by 25(OH) D status. There was no statistically significant difference in the mean age between participants with vitamin 25(OH) D deficiency compared to those without 25(OH) D deficiency (*p* = 0.1693). There was no statistically significant association between 25(OH) D status and gender (*p* = 0.5461), marital status (*p* = 0.3565), religion (*p* = 0.8656), education status (*p* = 0.2946), and occupation (*p* = 0.7519). None of the socio-demographic characteristics saw a significant association with vitamin D status(Table 1).

**Table 1:** Sociodemographic characteristics stratified by 25 (OH) D status

Characteristics	25(OH)D status		Statistic	*P*-value
	Deficient *N* = 218	Non-deficient *N* = 282		
Age	27.38 ± 6.28	28.42 ± 7.42	1.376^t^	0.1693
**Gender**			0.4085 ^x^	0.5461
Males	161(73.9)	201(71.3)		
Females	57(26.1)	81(28.7)		
**Marital status**			2.063 ^x^	0.3565
Single	141(64.7)	165(58.5)		
Married	73(33.5)	108(38.3)		
Divorced	4(1.8)	8(2.8)		
**Religion status**			0.2887 ^x^	0.8656
Christianity	162(74.3)	214(75.9)		
Moslems	43(19.7)	54(19.1)		
Traditionalist	13(5.9)	14(4.9)		
**Education**			3.710^x^	0.2946
Unschooled	28(12.8)	41(14.5)		
Basic	28(12.8)	48(17.0)		
Secondary	100(45.9)	107(37.9)		
Tertiary	62(28.4)	86(30.5)		
**Occupation**			1.205 ^x^	0.7519
Unemployed	6(2.8)	4(1.4)		
Informal	93(42.7)	124(43.9)		
Formal	27(12.4)	33(11.7)		
Student	92(42.2)	121(42.9)		

X: Chi−square test; t: unpaired t test; U: Mann−Whitney U Test. Values are presented as frequency (percentages), mean SD; Median (Interquartile ranges)

Table 2 shows the general characteristics of study participants. The average age of participants was 27.97 years. Male participants were significantly older compared to the female participant (*p* < 0.0001). There was no statistically significant difference in the median vitamin 25(OH) D (*p* = 0.8392), IL-10 (*p* = 0.5355), TNF-alpha (*p* = 0.9740) and IFN-gamma (*p* = 0.6908) between the males and females (Table 2).

**Table 2:** Cytokines and 25(OH)D characteristics stratified gender

Characteristics	Total (*n* = 500)	Males (*n* = 362)	Females (*n* = 138)	Statistics	*p*-value
Age	27.97 ± 8.36	29.50 ± 8.512	23.95 ± 6.454	6.933^t^	< 0.0001
25(OH)D (ng/ml)	25.05(7.82–41.78)	23.93(7.85–41.53)	26.05(7.74–41.92)	24,685^U^	0.8392
IL- 10 (pg/ml)	5.89(4.14–7.81)	5.98(4.20–7.81)	5.76(4.03–7.79)	24,083 ^U^	0.5355
TNF-alpha (pg/ml)	6.13(3.62–10.07)	6.09(3.62–10.47)	6.27(3.54–9.64)	24,931 ^U^	0.9740
IFN-gamma (pg/ml)	9.02(7.66–13.86)	9.03(7.63–13.64)	8.96(7.86–14.20)	24,403 ^U^	0.6908

t: unpaired t−test; U: Mann−Whitney U Test. Values are presented as frequency (percentages), mean SD; Median (Interquartile ranges)

Table 3 shows Spearman’s rho correlation of vitamin 25(OH) D with biochemical parameters among the general population. There was a statistically significant negative correlation between 25 (OH) D, TNF-alpha (*r*= -0.600; *p* < 0.0001), and IFN-gamma (*r*= -0.685; *p* < 0.0001). Meanwhile, there was a statistically significant positive correlation between 25 (OH) D and IL-10 (*r* = 0.448; *p* < 0.0001) (Table 3).

**Table 3:** Spearman’s rho correlation of vitamin 25(OH) D with biochemical parameters among the general population

Characteristics	25(OH)D (ng/ml) (*n* = 500)		*p*-value
	R	95% CI of r	
Age	0.028	-0.06262 to 0.1178	0.5347
IL-10 (pg/ml)	0.448	0.3724 to 0.5170	< 0.0001
TNF-alpha (pg/ml)	-0.600	-0.6551 to -0.5393	< 0.0001
IFN-gamma (pg/ml)	-0.685	-0.7297 to -0.6333	< 0.0001

r: Spearman’s rho regression; CI: confidence interval

Table 4 shows the diagnostics performance of biochemical markers for predicting 25(OH) D deficiency among the general population. The most accurate biochemical markers for identifying 25 (OH) D deficiency were IFN-gamma (AUC = 0.879; *p* < 0.0001) followed by TNF-gamma (AUC = 0.849; *p* < 0.0001) and IL-10 (AUC = 0.707; *p* < 0.0001). At a cut-off value of 9.75 pg/ml, sensitivity of 81.7%, specificity of 86.2%, PPV of 85.5% and NPV of 82.5% for IFN-gamma, 25 (OH) D deficiency was identified among study participants. At a cut-off value of 6.32 pg/ml for TNF-alpha, the sensitivity, specificity, PPV and NPV for identifying subjects with 25 (OH) D deficiency were 82.6%, 77.7%, 78.7% and 81.7% respectively. At a cut-off value of 5.59 pg/ml for IL-10, the sensitivity, specificity, PPV and NPV for identifying subjects with 25 (OH) D deficiency were 62.8%, 71.6%, 68.9% and 65.8% respectively (Table 4).

**Table 4:** Diagnostic performance of biochemical markers for predicting 25(OH) D deficiency among General Population

Biomarkers	AUC (95% CI)	Threshold value	Sensitivity	Specificity	PPV	NPV	Kappa
IL-10 (pg/ml)	0.707(0.662–0.753)*	5.59	62.8(56.3–68.9)	71.6(66.8–76.5)	68.9	65.8	0.678
TNF-alpha (pg/ml)	0.849(0.816–0.883)*	6.32	82.6(76.9–87.0)	77.7(72.4–82.1)	78.7	81.7	0.798
IFN-gamma (pg/ml)	0.879(0.847–0.911)*	9.75	81.7(75.9–86.2)	86.2(81.6–89.7)	85.5	82.5	0.842

PPV: positive predictive value; NPV: negative predictive value; CI: confidence interval; AUC: Area under the curve. * indicates significant AUC

Table 5 shows predictors of 25(OH) D deficiency using quartiles of biochemical parameters. At the 1st − 3rd quartile for IL-10 levels (Q1 = < 4.1pg/ml; Q2 = 4.1-5.9pg/ml; Q3 = 6.0-7.8pg/ml), participants had 22.04 times (p = < 0.0001), 4.76 times (*p* = 0.032) and 4.75 times (*p* = 0.040) increased odds of developing 25(OH) D deficiency respectively. At the 3rd and 4th quartile for TNF-alpha level (Q3 = 6.2-10.7pg/ml; Q4 = > 10.7pg/ml), participants had 68.29 times (*p* < 0.0001) and 187.37 times (*p* < 0.0001) increased odds of developing vitamin 25(OH) D deficiency respectively. At the 2nd– 4th quartile for IFN-gamma levels (Q2 = 7.7-9.0pg/ml; Q3 = 9.1-13.9pg/ml; Q4 = > 13.9pg/ml), participants had 6.00 times (*p* = 0.025), 63.02 times (*p* < 0.0001) and 146.35 times (*p* < 0.0001) increased odds of developing 25(OH) D deficiency respectively (Table 5).

**Table 5:** Predictors of 25(OH) D deficiency using quartiles of biochemical parameters

Parameter	AOR	95% CI	*P*-value
IL-10 (pg/ml)			
Q1(< 4.1)	22.04	4.34-112.06	< 0.0001
Q2(4.1–5.9)	4.76	1.15–19.71	0.032
Q3(6.0-7.8)	4.75	1.07–21.02	0.04
Q4(> 7.8)	1.0(reference)		
TNF-alpha (pg/ml)			
Q1(< 3.6)	1.0(reference)		
Q2(3.6–6.1)	2.99	0.51–17.71	0.227
Q3(6.2–10.7)	68.29	9.98–467.10	< 0.0001
Q4(> 10.7)	187.37	23.39-1501.02	< 0.0001
IFN-gamma (pg/ml)			
Q1(< 7.7)	1.0(reference)		
Q2(7.7-9.0)	6.00	1.25–28.89	0.025
Q3(9.1–13.9)	63.02	11.2-353.42	< 0.0001
Q4(> 13.9)	146.35	24.04-890.08	< 0.0001

Q1 = 1st quartile; Q2 = 2nd quartile; Q3 = 3rd quartile and Q4 = 4th quartile. Age adjusted odds ratio

## Discussion

This study explored the contrary effect on pro and anti-inflammatory the effect vitamin D has on the pro-inflammatory cytokines (TNF-alpha, IFN-gamma) and anti-inflammatory cytokine (IL 10) among apparently healthy Ghanaian study participants. Considerable attention has been shown lately to the immunomodulatory influence of vitamin D and the role it plays in the normal functioning of the body. While the production of vitamin D (1,25OHD) as an immunomodulatory effector depends on 25OHD (the inactive form) which is the major circulating form of vitamin D or pro-inflammatory cytokines such as IFN-γ, IL-6 or TNF-α induction, the interplay between vitamin D status, these inflammatory cytokines, and immune cells in supposedly sufficient sunshine environment has not been evaluated.

The study determined the interplay between some immunological cytokines (IL 10, TNF-alpha and IFN-gamma) and their association with vitamin D status among the Ghanaian populace. The results of the study confirmed a constructive association between vitamin D deficiency and IL 10 and a negative correlation between vitamin D deficiency, TNF-alpha, and IFN-gamma. There was however no statistically significant difference in the median 25(OH) D (*p* = 0.8392), IL-10 (*p* = 0.5355), TNF-alpha (*p* = 0.9740) and IFN-gamma (*p* = 0.6908) between the males and females (Table 2). There was no statistically significant association between 25(OH) D status, the mean age (*p* = 0.1693) and gender (*p* = 0.5461) (Table 1).

Various inflammatory diseases have been associated with vitamin D deficiency and a study suggested that vitamin D inhibits monocyte pro-inflammatory cytokines like TNF-alpha and IFN-gamma induction by increasing the production of anti-inflammatory cytokine like IL10 [20]. Some studies have demonstrated negative correlation between pro-inflammatory cytokines like TNF-alpha, IFN-gamma and 25(OH) D, and a positive association between IL10 and vitamin 25(OH) D [12, 21–23].

The findings in this current study support previous findings in that there was a significant positive link between IL10 and 25(OH) D and a significant negative association between TNF-alpha, IFN-gamma and 25(OH) D (Table 3). This is an indication that participants with vitamin D deficiency had significant increased pro-inflammatory cytokines (TNF-alpha and IFN-gamma) levels because the anti-inflammatory cytokine (IL10) levels were significantly reduced to be able to inhibit the pro-inflammatory cytokines levels hence the results seen in low vitamin D participants unlike participants with the optimal vitamin D who had significantly increased IL10 and reduced pro-inflammatory cytokines levels (Table 2).

This study was therefore in accordance with most literature and did not contradict already established findings and associations between 25(OH) D, IL10, TNF-alpha and IFN-gamma. The potential mechanism underlying these associations has been linked to the fact dendritic cells, macrophages, T and B cells being immune cells express vitamin D and 1-α-hydroxylase (CYP27B1), the vitamin D activating enzyme which plays an important role in inflammation [3, 8].. Non-immune cells have also been reported to express vitamin D and its activating enzyme. Previous studies have indicated that vitamin D is a pivotal regulator of immune function and inflammation and it is very essential in preventing some cancers and inflammatory disorders [3]. An observational study of healthy women from the University of Missouri-Columbia campus recorded an inverse relationship between D 25(OH)D and TNF-alpha concentrations but did not witness any significant positive correlation between D 25(OH)D and IL10 [22]. The present study was consistent with their findings that had an inverse relationship between D 25(OH)D and TNF-alpha concentrations but saw a direct association between vitamin D 25(OH)D and IL10 (Table 3). This findings in this study are consistent with the findings in cross-sectional study among healthy adult Japanese where a positive association of serum vitamin D 25(OH)D with anti-inflammatory cytokines and a negative correlation with IFN-gamma [24] which was established.

A seasonal study on cytokine regulation responses of vitamin D status in healthy individuals saw a significant rise in both 25(OH)D and vitamin D 1,25(OH)D concentrations but significantly decreased levels of IL10, TNF-alpha, and IFN-gamma during summer [25]. This was not in consistent with our current findings where we had a direct association of IL10 with vitamin D. Another contrast study also reported 1,25(OH)_2_D_3_ inhibiting both IFN-γ and IL‐10 in CD4^+^ T lymphocytes [26]. Again a study also reported lipopolysaccharide-induced TNF-alpha inhibition by 1,25(OH)_2_D_3_ which was similar to this current findings [27]. Matilainen et al. in their study also reported impaired IL‐10 production induced by 1,25(OH)_2_D_3_ but attributed the reason to short-term down-regulation [28] which was also in contrast to our current findings. A clinical trial study did not show an effect of vitamin D supplementation on the changing in both pro-inflammatory and anti-inflammatory cytokines levels [29]. Out of seven (7) systematic reviews done by Calton et al., four (4) had reports of vitamin D having anti-inflammatory effect, one (1) had mixed effect and two (2) reported pro-inflammatory effect [30].

Most studies on the association between vitamin D status and cytokines in Africa have not been done among apparently healthy individuals. Those studies done among non-healthy individuals have also reported similar findings in this current study. A cross-sectional comparative study relationship between serum 25-hydroxyvitamin D and inflammatory cytokines in paediatric sickle cell disease in Nigeria saw that vitamin D supplementation lowered inflammatory and improved anti-inflammatory cytokines in sickle cell disease management [31].

There is abundance of sunshine during summer just as sunshine is witnessed throughout the year in Ghana. The major source of vitamin D has been shown to be from the body’s exposure to sunlight and it is assumed that Ghanaians are expected to have optimal vitamin D for normal body function, but the body vitamin D levels did not correlate very well with the amount of sunlight in the study region. The explanation of low vitamin D levels in healthy individuals which led to the pro-inflammatory cytokines levels (TNF-alpha and IFN-gamma) being significantly increased, whiles anti-inflammatory cytokine level (IL10) was notably reduced could not be explained in this present findings. Though there could be underlying reason for the cause, further studies need to be conducted to include more cytokines and also on vitamin D receptor genes since studies at the genetic level could be of great importance and bring out better explanation.

The diagnostics performance of biochemical markers for predicting 25(OH) D deficiency among the general population were determined (Table 4). The most accurate biochemical markers for identifying vitamin 25 (OH) D deficiency were IFN-gamma (AUC = 0.879; *p* < 0.0001) followed by TNF-alpha (AUC = 0.849; *p* < 0.0001) and IL-10 (AUC = 0.707; *p* < 0.0001). Quartiles of biochemical parameters of the cytokines in predicting vitamin 25OHD deficiency were also determined. At a lower quartile for IL 10, participants had higher odds of developing 25(OH) D deficiency. In other words, the higher the quartile value for IL10, the less risk of developing deficiency.

At higher quartiles (meaning at higher values) for both TNF-alpha and IFN-gamma, participants had higher risk of developing vitamin 25(OH) D deficiency. Though both TNF-alpha and IFN-gamma production is induced by vitamin D in response to inflammatory reaction, their activity could cause tissue damage and in that regard, vitamin D equally induces the production of anti-inflammatory cytokine to regulate their activity hence their normal levels in people without vitamin D deficiency and their higher risk of deficiency in large quantity (Table 5).

### Limitation of study

The study had some limitations. The study intended to use healthy population in view of that, standard criteria and donor selection screening were used to select the participants as apparent healthy donors since the study could not do all comprehensive test diagnostic analysis to diagnose someone as being very healthy. Any unknown underlying disease condition in some very few participants which could not be checked is likely to influence the findings. The study again did not measure body mass index of participants with vitamin D levels and the measured cytokines to see their association. Despite limitations, the study provides valuable insights of the relationship of vitamin D and pro and anti-inflammatory cytokines among healthy people in the Ghanaian population.

## Conclusion

There was a significant association between vitamin D levels and pro-inflammatory cytokines (TNF-alpha, IFN-gamma) and anti-inflammatory cytokine (IL 10) among healthy Ghanaian populace. The results of the study is an indication of a possible role of vitamin D in contributing to balancing cytokines towards an anti-inflammatory role in inflammatory situations among healthy people.

## Data Availability

The data presented in the results of this study will be made available on reasonable request to Dr Tonnies Abeku Buckman. E-mail address: tonniesb@yahoo.com.

## Abbreviations

Vitamin 25(OH)D 25: hydroxycholecalciferol
TNF: Tumor necrosis factor
IFN: Interferon
IL: Interleukin
ELISA: Enzyme linked immunosorbent assay
AUC: Area under the curve
PTH: Parathyroid hormone
*VDR*: Vitamin D receptor gene
1,25(OH)2D: 1, 25-dihydroxyvitamin D
CYP27B1: Cytochrome P450 family 27 subfamily B member 1
Th1: Type 1 helper cells
Th2: Type 2 helper cells
AR: Ashanti Region
UWR: Upper West Region
NR: Northern Region
BA: Brong-Ahafo
UER: Upper East Region
WR: Western Region
ER: Eastern Region
STIs: Sexually transmitted infections
K_2_EDTA: Dipotassium ethylene diamine tetra acetic acidic
